# Comparison of the Efficacy and Safety of Two Cryotherapy Protocols in the Treatment of Common Viral Warts: A Prospective Observational Study

**DOI:** 10.1155/2020/2309309

**Published:** 2020-07-13

**Authors:** Jihan M. Muhaidat, Firas A. Al-qarqaz, Diala M. Alshiyab, Hadeel S. Alkofahi, Yousef Khader, Mawaddah Y. Ababneh

**Affiliations:** ^1^Department of Dermatology, Faculty of Medicine, Jordan University of Science and Technology, Irbid, Jordan; ^2^Department of Public Health, Community Medicine, Jordan University of Science and Technology, Irbid, Jordan

## Abstract

**Background:**

Cryotherapy (freezing by liquid nitrogen) is an effective and widely used method for treatment of common warts. Patients often need multiple sessions at variable intervals. Protocols used by different dermatologists vary in terms of freezing time, the number of cycles, and the intervals between sessions.

**Aim:**

To compare the efficacy (cure rates) and safety (complications, early and late) of two cryotherapy treatment protocols for common viral warts.

**Method:**

A prospective observational study was conducted; it involved 80 patients with common warts on the hands and feet who were treated with cryotherapy done by two dermatologists who use different protocols; group 1 (45 patients) were treated by a single cycle of 10 seconds of freezing at 2 weekly intervals, and group 2 (35 patients) received a single cycle of 20 seconds of freezing at 4-weeks intervals. The two protocols were compared in terms of cure rate and complications 1-2 months after the final treatment. Recurrence rate and late complications were assessed at 9–12 months after the final treatment.

**Results:**

Group 1 patients achieved higher cure rate than group 2, 77.8% and 54.3%, respectively (*P*=0.001). Early (blistering) and late complications (dyspigmentation and scarring) were comparable in both groups. Pain score associated with protocol 1 (5.2/10) was less than protocol 2 (6.4/10) (*P*=0.004). Recurrence rate (17%) was comparable in both groups. Association between cure rate and duration of warts (*P*=0.022) and also association between cure rate and the mean number of warts (*P*=0.001) were demonstrated.

**Conclusions:**

Cryotherapy is an effective and safe treatment for common viral warts of hands and feet. The impact of shorter intervals on cure rate was more significant than increasing freezing time with longer intervals between freezing sessions. The study was approved by the local IRB committee (285-2018).

## 1. Introduction

Warts are caused by keratinocyte infection with human papilloma virus. Verruca vulgaris (common wart) is the most frequent type commonly seen on the hands. Disfigurement and concerns about spreading warts to self and close contacts can cause significant embarrassment to patients; this encourages many patients to seek active treatment rather than waiting for spontaneous clearance [1]. While warts may resolve spontaneously within a year in 50% of children, they tend to persist for years in adults [2–4].

Cryotherapy is an established, generally safe, and simple method in the treatment of warts. It can be offered as a first- or second-line treatment, especially for patients with few warts of short duration. Freezing by liquid nitrogen, the most commonly used cryogen, can be delivered by cotton wool buds and more frequently by a cryospray gun. The best outcome can be achieved by conducting the correct technique by a skilled operator, while always addressing contraindications and side effects [5, 6].

Cure rate attained by cryotherapy has been varied between trials with a mean of 49% (3); this can be attributed to many factors, which include among others: the age, immunity of the patient, wart type, and duration of warts [7–9]. Paring thick keratotic warts can improve efficacy of cryotherapy [8, 10]. Freezing time and the number of freeze-thaw cycles, as well as the interval between sessions, are additional variables that can impact the cure rates. Overall, it seems that more aggressive and frequent sessions are associated with higher cure rate; however, risk of side effects, especially pain and blistering, is higher [3, 5].

The main objective of this study is to assess the efficacy of two cryotherapy treatment protocols: a sustained (10 seconds) freezing time at 2 weekly intervals, compared to more aggressive (20 seconds) sessions at 4 weekly intervals. A secondary objective of the study is to evaluate the safety of both treatment protocols in terms of risk of pain, blistering, dyspigmentation, and scarring.

## 2. Materials and Methods

### 2.1. Setting

This study was conducted at dermatology clinics of King Abdullah University Hospital, Irbid, Jordan, over the period from June 2018 to December 2019. The study was approved by the local IRB committee (285-2018).

### 2.2. Patients

Patients considered as potential participants in this study included those who were 12 years or older with common viral warts on the limbs (upper and lower limbs), whose dermatologist assessed as eligible for cryotherapy, not using any treatment for at least 1 month prior to inclusion in the study. The following patients were excluded: patients on combination therapy and those previously treated by cryotherapy, wart type other than the common type including warts that needed paring prior to cryotherapy.

All potential patients/guardians were counselled about the study protocol and possible side effects, and those willing to participate were asked to sign informed consent prior to enrolment.

Baseline demographic data, medical history relevanct and comorbidities, duration of warts, their number and site were recorded at baseline visit.

### 2.3. Study Design

This was a prospective observational study in which patients treated by two dermatologists who adopt different protocols of cryotherapy were divided into two groups; group 1 included 45 patients who received cryotherapy sessions of 10 seconds of freezing fortnightly, and group 2 included 35 patients who received sessions of 20 seconds of freezing monthly. Patients were treated for a maximum of 3 months, so patients in groups 1 and 2 could receive a maximum of 6 or 3 sessions, respectively. Wart site was examined for cure, dyspigmentation, or scarring at each session. If warts were cured, no cryotherapy was provided, and the patient was brought 1-2 months later for final assessment. Final assessment (primary end-point assessment) for efficacy and side effects was done 1-2 months after the last treatment. Patients were followed up for 9–12 months after the last treatment to assess for recurrence and long-term sequelae. Cure was defined as disappearance of warts (resolution of roughness and black dots) based on clinical examination including dermoscopic evaluation for doubtful cases.

Cryotherapy was delivered by a spray gun using liquid nitrogen (CRY-AC-3, Brymill Cryogenic system), directly sprayed onto the wart through nozzle D, which was appropriate for all warts treated. The nozzle was held 1-2 cm from the skin surface, and the cryogen was sprayed in the centre until an ice ball forms that completely encompasses the wart; spraying was sustained for 10 or 20 seconds depending on the protocol used.

After each treatment, patients were asked to assess pain severity by the visual analogue scale (VAS), 0–10, where 0 is no pain, and 10 is the worst pain ever.

### 2.4. Outcome

The primary outcome is the efficacy of the protocol as measured by cure rate, defined as complete disappearance of all warts by clinical and dermoscopic assessment, 1-2 months after the final treatment. The secondary outcomes were the pain score and the local side effects including blistering, dyspigmentation, and scarring.

### 2.5. Statistical Analysis

Statistical analysis was performed using IBM SPSS software version 24 (IBM SPSS Statistics, Armonk, NY). Multivariate analysis was conducted to determine the difference in the cure rates between the two groups after adjusting for significant variables. Odds ratios were calculated using logistic regression. A *P* value <0.05 was considered statistically significant.

## 3. Results

Eighty patients (35 males and 45 females) with common warts on the hands and feet were recruited into this study. Their age ranged from 12 to 66 years with a mean age (SD) of 27.7 (12.7) years. The two groups had comparable demographic and clinical characteristics as illustrated in Table 1. However, patients in group 2 were slightly older, and the duration of their warts was longer, but the differences were not statistically significant.

Sixty seven of the eighty patients have completed the study; the overall cure rate for participants in both groups was 67.5% (intention-to-treat analysis (ITT)) at final assessment 1-2 months after the last treatment. The results according to the protocol are illustrated in Table 2.

Group 1 (10 seconds, *Q* 2 weeks) treatment protocol was more effective in achieving cure compared with group 2 (20 seconds, *Q* 4 weeks), and the difference was statistically significant. The cure rate based on intention-to-treat analysis (ITT) was 77.8% for group 1 (35/45) and 54.3% for group 2 (*P*=0.026). Cure rate calculated based on per protocol (PP) analysis shows higher cure rates for both groups, (95%) (35/37) for group 1 and (63%) (19/30) for group 2 (*P*=0.001). In addition, 86% of the total number of warts (113) treated by protocol 1 had been cleared at the end of the three months, while 69% of the warts (84) treated by protocol 2 had been cleared (*P*=0.038).

The power of the study to detect a difference of approximately 25% in the cure rate between the two groups using a sample size of 80 subjects exceeded 80% at a level of significance of 0.05.

**Figure 1:**
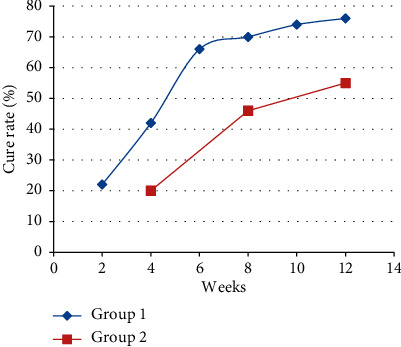
Cure rates of treatment groups over time.

The mean number of sessions to cure was 2.5 sessions in protocol 1 compared with 1.8 sessions for protocol 2; this corresponded to 5 and 7.2 weeks for protocols 1 and 2, respectively (*P*=0.054). Interestingly, cure rate over time showed superiority of the group 1 protocol although the cure rate after the first session was almost equal for both treatment groups as shown in Figure 1.

The impact of other factors on cure rate was also studied. Duration of warts had significant impact; in fact, increased duration by one month was associated with decreased odds of cure by 5%. After adjusting for the duration of the disease, patients in protocol 1 were significantly more likely to achieve cure compared with those treated by protocol 2 (OR = 2.7, 95% confidence interval: 1.1–7.4; *P*=0.022).

An association between the mean number of warts and cure was found (*P*=0.001). Patients who achieved cure from both groups had a mean number of warts of 1.8, while patients who failed the treatment had a mean number of warts of 3.7; it is unlikely that the difference in efficacy between the two protocols is affected by this finding as the mean number of warts for both groups was similar.

Regarding secondary outcomes, the pain intensity elicited by protocol 1 was less than that by protocol 2; the overall average pain score for patients treated with protocol 1 was 5.2/10, while it was 6.4/10 for protocol 2; this difference was significant (*P*=0.004).

Both protocols of cryotherapy were tolerated well; the difference in side effects was not statistically significant between the two groups. Blisters were reported by 21% and 31% of patients on protocols 1 and 2, respectively, and all blisters healed within a few days. At their final assessment, 18% of patients on protocol 1 and 29% of patients on protocol 2 had hypopigmentation. Few patients developed long-term consequences at 9–12 months; about 3% and 2% of patients on protocol 1 had hypopigmentation and hyperpigmentation, respectively, while for patients on protocol 2, 2.5% had hypopigmentation and 5% had hyperpigmentation. None of the patients developed scarring.

The overall recurrence rate of warts after a period of 9–12 months from the last treatment for both groups was 17%, and patients on protocol 1 had a recurrence rate of 14%, while it was 21% for those on protocol 2 (*P*=0.532).

## 4. Discussion

This study is the first to compare two protocols of cryotherapy that differ in two factors, freezing time and the interval between sessions; both have impact on the outcome as short interval and longer freezing time may enhance efficacy.

Bunney et al. in 1976 concluded that conducting cryotherapy at 4-weeks interval led to significant reduction in the percentage of cure in comparison to 2 weeks and 3 weeks of interval [11]. Furthermore, two studies that compared treatment intervals concluded that 2 weekly intervals are the optimal regarding efficacy and safety as it allows a greater number of sessions to be delivered within a specified period and is usually enough for healing of blisters and soreness before the forthcoming session. It was also associated with less recurrence when compared with 3 weekly sessions [10, 12].

Warts may be frozen for a duration of 5–30 seconds; this upper limit should not be exceeded to decrease the risk of scarring and depigmentation especially in colour of skin [6, 13]. Previous studies had concluded that aggressive 10 seconds of freezing was more effective than gentle freezing but was associated with more morbidity [14–16]. Double versus single freeze-thaw cycle at 3-weeks interval found a beneficial value for planter warts but not for common hand warts [17].

The results of this study indicate that protocol 1 of cryotherapy (10 seconds, *Q* 2 weeks) was more effective and rapid in achieving cure than protocol 2 (20 seconds, *Q* 4 weeks). This shows that, in this cohort of patients, performing treatment at shorter intervals was the most important factor in curing warts. Not only this, when sessions were done at longer interval (4 weeks), this resulted in a significant drop in cure rates despite increasing the freezing time (in this case, doubling time from 10 to 20 seconds). In addition, protocol 1 was better tolerated in terms of associated pain, while side effects including blistering, scarring, and dyspigmentation, along with the recurrence rate, were comparable for both groups of patients.

Conducting cryotherapy treatment at less frequent intervals may be preferred by some patients and has the advantage of decreasing workload on busy dermatology and primary care clinics; however, doubling the freezing time at 4-week interval did not lead to comparable cure rates at 2 weeks. Enhanced efficacy (around 25% increase in cure rate) has been actually accomplished by conducting treatments of cryotherapy of 10 seconds at 2 weeks, and this was not compensated for by doubling the freezing time at 4-weeks interval.

The tendency of warts of shorter duration for a more favourable outcome revealed by this study is in agreement with results obtained from previous studies [8, 9]. Another important observation in this study is that patients with fewer number of warts were more likely to achieve higher cure rates. This has also been shown in other studies [4, 10]; these findings may favour early initiation of treatment over the option of waiting for spontaneous clearance of warts.

The side effects including early and late complications were comparable in both groups. There were slightly more blistering and hypopigmentation in patients treated by protocol 2 (20 seconds, 4 weekly), although this difference did not reach statistical significance. Only few patients from both groups had long-term consequences in the form of dyspigmentation. These findings generally point to good safety profile for cryotherapy, especially with nonaggressive treatment times.

The recurrence rate of warts in this cohort was 17% for both groups (14% for protocol 1 and 21% for protocol 2). In the literature, the reported recurrence rates vary between 16.7% and 35%, but it would be difficult to make comparisons as different treatment protocols were used [10].

## 5. Conclusion

Cryotherapy is an effective treatment for common viral warts. Freezing for 10 seconds every 2 weeks was associated with higher cure rates and lower recurrence compared with 20 seconds of freezing at a longer interval of 4 weeks. Patients with fewer warts and/or less duration were more likely to achieve higher cure rates. The side effects, both early and late, were comparable in both treatment groups; however, pain was significantly less severe with less freezing duration.

## Figures and Tables

**Table 1:** The demographic and clinical details of both treatment groups.

Variable	Group				*P*‐value
	1 (10 seconds/2 weeks)		2 (20 seconds/4 weeks)		
	*N*	(%)	*N*	(%)	
Gender					0.608
Male	18	40	16	45.7	
Female	27	60	19	54.3	
Age, mean (SD)	25.8 (10.9)		30.4 (14.5)		0.107
Number of warts, mean (SD)	2.5 (2.2)		2.4 (2.1)		0.817
Duration of warts in months, mean (SD)	8.7 (10.8)		12.4 (15)		0.207
Site of warts					0.371
Hand	36	80	24	68	
Foot	6	13.3	9	25.7	
Both	3	6.7	2	5.7	

**Table 2:** Response to treatment (cure and side effects) in both treatment groups.

	Protocol 1 *N* = 45		Protocol 2 *N* = 35		*P*‐value
	*n*/*N*	(%)	*n*/*N*	(%)	
Cure rate (out of the total number) (intention to treat (ITT))	35/45	77.8	19/35	54.3	0.001
Cure rate (out of the number who completed the study) (per protocol (PP))	35/37	94.6	19/30	63	0.026
Number of warts cleared	91/113	86	55/84	69	0.038
Mean number of sessions to cure	2.5		1.8		0.054
Average pain score	5.2		6.4		0.004
Blisters	9/45	21	11/35	31	0.247
Hypopigmentation	8/45	18	10/35	29	0.251
Recurrence	5/35	14	4/19	21	0.532

## Data Availability

The data used to support the findings of this study are available from the corresponding author upon request.
